# Vitamin D, parathyroid hormone and metabolic syndrome – the PORMETS study

**DOI:** 10.1186/s12902-017-0221-3

**Published:** 2017-11-17

**Authors:** Luís Raposo, Sandra Martins, Daniela Ferreira, João Tiago Guimarães, Ana Cristina Santos

**Affiliations:** 1 Grupo de Estudo da Insulino-Resistência, Sociedade Portuguesa de Endocrinologia, Diabetes e Metabolismo, Lisboa, Portugal; 2 EPIUnit - Instituto de Saúde Pública da Universidade do Porto (ISPUP), Porto, Portugal; 3 Serviço de Patologia Clínica, Centro Hospitalar de S. João, Porto, Portugal; 4 Departamento de Biomedicina, Faculdade de Medicina, Universidade do Porto, Porto, Portugal; 5 Departamento de Ciências da Saúde Pública e Forenses e Educação Médica, Faculdade de Medicina, Universidade do Porto, Porto, Portugal

**Keywords:** Vitamin D, PTH, Functional hypoparathyroidism, Metabolic syndrome, Cardiovascular risk, Prevalence, Portugal

## Abstract

**Background:**

Vitamin D (VitD) and parathyroid hormone (PTH) play important roles in calcium metabolism and skeletal homeostasis. Estimates of the VitD status in several European countries show large variations between them. In addition, no national population-based estimate has been published. VitD and PTH may also play important roles in cardiovascular risk, which has been suggested to be associated with metabolic syndrome (MetS) and is very prevalent in Portugal.

The goal of our study was to evaluate the prevalence of hypovitaminosis D and its determinants as well as PTH serum level determinants and associations of the 25-hydroxyvitamin D and PTH serum levels with MetS and its individual components in a sample of the Portuguese mainland population.

**Methods:**

PORMETS is a national cross-sectional study that includes a total sample of 4095 adults. A subsample, including 500 participants, was randomly selected for the present study. A structured questionnaire was administered to collect information on personal medical histories and socio-demographic and behavioral characteristics. Blood pressure and anthropometrics measurements were performed. Fasting venous samples were collected and PTH and 25-hydroxyvitamin D were measured. VitD adequacy was classified according to the Institute of Medicine, and MetS was classified according to the Joint Interim Statement recommendations. Multiple linear regression and unconditional logistic regression models were used to estimate the associations between the levels of PTH and 25-hydroxyvitamin D and with MetS and its individual components.

**Results:**

The prevalence of VitD deficiency was 37.7%, and MetS was present in 191 participants (38.4%). The serum PTH levels showed a positive association (OR: 1.014; 95%CI: 1.002, 1.026) with the waist circumference component of MetS. The serum 25-hydroxyvitamin D levels were negatively associated with MetS (OR: 0.957; 95%CI: 0.922, 0.993) as well as with its blood pressure (OR: 0.949; 95%CI: 0.912, 0.987) and triglycerides (OR: 0.930; 95%CI: 0.892, 0.969) components.

**Conclusion:**

This study showed a high national prevalence of hypovitaminosis D. The PTH levels showed a significant positive association with the WC component of MetS, and the VitD levels were negatively associated with the BP and triglycerides components as well as with the MetS.

## Background

Vitamin D (VitD) is a fat-soluble vitamin that is involved in the metabolism of calcium and skeletal homeostasis [1]. Cholecalciferol (VitD3), the main source of VitD, is synthesized in the skin from the cholesterol precursor 7-dehydrocholesterol through exposure to ultraviolet (UV) B radiation. VitD from dietary sources and sun exposure is not biologically active and must undergo two hydroxylations in the human body for activation. VitD is first hydroxylated by the liver to form 25-hydroxyvitamin D [25(OH)D], also known as calcidiol, which is then primarily hydroxylated by the kidney to form the physiologically active 1α,25(OH)_2_D, or calcitriol. Calcidiol has low bioactivity but is the main form of VitD in the blood stream and best indicator of VitD status*.*

According to a recent review [2], estimates of the VitD status in several European countries showed large variations. No national population-based study has been published, and the only available Portuguese studies involved regional or specific groups using hospital-based recruitment. According to a recent study in Portuguese hospitalized patients, VitD deficiency or inadequacy was present in 60.3% of these patients [3].

An inadequate VitD status may play a significant role in cardiovascular disease (CVD) risk [4], and several observational studies suggest an association between hypovitaminosis D and metabolic syndrome (MetS), which represents a cluster of interrelated risk factors for CVD [5]. In addition, MetS is highly prevalent in Portugal. [6] An evaluation of the potential associations of MetS and its individual components with VitD levels may support and enhance current knowledge of the effects of VitD on CVD risk factors.

Parathyroid hormone (PTH), a hormone with a close regulatory relationship with VitD, has also been associated with CVD events [7]. Findings from population-based cross-sectional studies have suggested a positive association between PTH and MetS among older men [8] and in morbidly obese individuals [9]. The goal of our study was to evaluate the prevalence of hypovitaminosis D and its determinants as well as PTH serum levels determinants and associations of the 25(OH)D and PTH serum levels with MetS and its individual components in a sample of the Portuguese mainland population.

## Methods

PORMETS (PORtuguese METabolic Syndrome) is a national cross-sectional study that includes a sample of adults registered in primary health care centers of the Portuguese mainland. Information regarding PORMETS recruitment proceedings and methodology have been previously published by the authors [10]. According to national legislation, all citizens are enrolled in the health center of their zone of residence. In each of the eighteen Portuguese mainland administrative regions (districts), two health care centers were included. One was in the district’s capital and the other represented a non-urban area. In each center, participants were randomly selected from the general practitioners’ lists, and 120 participants were evaluated under an inclusion criterion of 18 years of age or older. The selected participants went to the health center specifically to participate in the study. A total of 4105 participants were evaluated, and information was collected from February 2007 to July 2009. Ten participants were excluded from data analysis because they were pregnant at the time of the interview. Therefore, 4095 participants remained.

For this particular study, 500 participants (286 women and 214 men) were randomly selected from the initial PORMETS sample. This sub-sample size was calculated considering a margin of error of 5%, confidence level of 95% and response distribution of 50% for the proportion of participants with 25(OH)D levels below 30 ng/mL (75 nmol/L). The comparison between the selected and unselected participants did not show significant differences, except for systolic blood pressure and for insulin serum levels (Table 1). The PORMETS study was approved by the Portuguese Regional Health Administrations, the Ethics Committee of the São João Hospital E.P.E. and the Portuguese Data Protection Authority. Additionally, approval from each Clinical Director of the health care centers was received, and all participants provided written informed consent.

**Table 1:** Comparison between selected and unselected participants

Variables	Unselected participants	Selected participants	*p* value^a^
Gender (n)			
Women	2069	286	
Men	1526	214	0.881
Age – years [median (P25, P75)]	54 (41, 66)	53 (41, 67)	0.856
Education level – years [median (P25, P75)]	4 (4, 10)	6 (4, 10)	0.252
Alcohol intake (n)			
Non-drinker	1618	229	
Former drinker	124	18	
Occasional drinker	934	128	
Daily drinker	892	123	0.990
Smoking habits (n)			
Non-smoker	2486	358	
Former smoker	521	66	
Smoker	529	70	0.597
Physical exercise (n)			
No	2592	352	
Yes	954	139	0.597
UV exposure (n)			
Lower	1971	283	
Higher	1555	217	0.768
Weight – cm [median (P25, P75)]	71.0 (62.5, 80.5)	71.0 (61.0, 80.0)	0.704
Height – cm [median (P25, P75)]	162.0 (155.5, 169.0)	162.0 (155.0, 169.0)	0.745
BMI – Kg/m^2^ [median (P25, P75)]	27.0 (24.0, 30.0)	27.1 (24.1, 29.8)	0.813
WC – cm [median (P25, P75)]	93.4 (85.0, 101.5)	93.5 (86.0, 102.0)	0.399
Systolic BP – mmHg [median (P25, P75)]	130 (116, 145)	131 (119, 147)	0.038
Diastolic BP – mmHg [median (P25, P75)]	79 (70, 86)	80.0 (70, 87)	0.073
Glucose – mg/dL [median (P25, P75)]	85 (77, 97)	85 (77, 97)	0.947
Insulin - μU/mL [median (P25, P75)]	7.6 (5.1, 11.4)	8.0 (5.3, 12.2)	0.040
HOMA – median (P25, P75)	1.6 (1.0, 2.6)	1.7 (1.1, 2.9)	0.119
hs-CRP - mg/L [median (P25, P75)]	0.15 (0.07, 0.36)	0.16 (0.08, 0.39)	0.626
Cholesterol – mg/dL [median (P25, P75)]	206 (179, 233)	204 (180, 233)	0.611
Triglycerides – mg/dL [median (P25, P75)]	106 (78, 147)	104 (76, 143)	0.242
HDL-cholesterol – mg/dL [median (P25, P75)]	47 (39, 55)	47 (39, 55)	0.553
MetS (n)			
No	2086	307	
Yes	1403	191	0.428
BP component (n)			
No	1352	177	
Yes	2172	322	0.212
WC component (n)			
No	1812	253	
Yes	1702	247	0.686
Glycemia component (n)			
No	2655	382	
Yes	822	112	0.635
HDL-cholesterol component (n)			
No	1566	224	
Yes	1935	276	0.977
Triglycerides component (n)			
No	2615	383	
Yes	878	117	0.401

*SD* standard deviation, *UV* ultraviolet, *BMI* body mass index, *WC* waist circumference, *BP* blood pressure, *HOMA* homeostatic model assessment, *hs-CRP* high sensitivity C-reactive protein, *MetS* metabolic syndrome

^a^Chi-square test/*Fisher*’s exact test or Mann-Whitney U test *p* value

A structured questionnaire was administered to collect information on personal medical histories and socio-demographic and behavioral characteristics. The participants were considered current smokers if they smoked daily or occasionally, former smokers if they had stopped smoking for at least 6 months, and non-smokers if they had never smoked. Regarding alcohol intake, participants were categorized as occasional drinkers if they had less than one drink per day, daily drinkers if they consumed at least one drink per day, former drinkers if had stopped drinking for at least 6 months and non-drinkers if they had never consumed any type of alcoholic beverage. Regular physical exercise was considered when the participant was engaged in some leisure time physical activity on a repeated basis for at least 30 min a week. Participants who were evaluated in the June–November period and December–May period were classified, respectively, as “higher” and “lower” UV radiation exposure.

Anthropometrics measurements were performed, including weight, height (Ht) and waist (WC) and hip (HC) circumferences. Weight was measured to the nearest 0.1 kg using a digital scale, and Ht was measured to the nearest centimeter in the standing position using a wall stadiometer. WC was measured midway between the bottom of the rib cage and iliac crest, and HC was measured as the maximum circumference of the buttocks. The waist-to-height ratio (WHtR) was calculated as the WC divided by the Ht, and the waist-to-hip ratio (WHR) was calculated as the WC divided by the HC. Body mass index (BMI) was calculated as weight in kilograms divided by the square of Ht in meters, and participants were classified according to the World Health Organization criteria [11]: underweight, normal range, pre-obese and obese categories, which are defined as BMI < 18.5 kg/m^2^, ≥18.5 to <25 kg/m^2^, ≥ 25 to <30 kg/m^2^ and ≥ 30 kg/m^2^, respectively.

Blood pressure (BP) was measured on a single occasion using a standard mercury sphygmomanometer with the cuff on the right upper arm after a 10-min rest. Two BP readings were taken and the mean of the two readings was calculated. If the difference between the two measurements was greater than 5 mmHg for the systolic or the diastolic BP, a third measurement was taken and the mean of the two closest values was registered.

Fasting venous blood samples were collected by trained nurses in each health care center, and the samples were stored at −80 °C.

A chemiluminescent immunoassay using a Liaison automated analyzer (Diasorin Iberia, Madrid, Spain) was used to measure 25(OH)D. Biointact PTH and insulin were determined by an electro-chemiluminescent immunoassay using a Cobas e411 automated analyzer (Roche, Amadora, Lisboa, Portugal). High sensitivity C-reactive protein (hs-CRP) was measured using a particle-enhanced immunonephelometric assay on a BN^®^II laser nephelometer. (Siemens Healthcare, Amadora, Lisboa, Portugal). All other parameters (glucose, total cholesterol, triglycerides, HDL-cholesterol, calcium, phosphorus, albumin and creatinine) were measured using conventional methods with an Olympus AU5400^®^ automated clinical chemistry analyzer. (Beckman-Coulter^®^,Oeiras, Lisboa, Portugal). Insulin resistance was estimated by the Homeostatic Model Assessment (HOMA), from fasting glucose (mmol/L) and insulin (μUI/mL), as the product of the two divided by 22.5.

VitD adequacy was classified according to the Institute of Medicine (IOM) recommended cut-off values for 25(OH)D levels [12]: deficiency below 12 ng/mL (30 nmol/L); inadequacy ≥ 12 and <20 ng/mL (≥ 30 and <50 nmol/L) and sufficiency ≥ 20 ng/mL (≥ 50 nmol/L). Hyper- and hypoparathyroidism were defined as PTH levels above and below the standard laboratory reference range (10–65 pg/mL), respectively, and a “blunted PTH response” was defined as a PTH level within the reference range in the presence of 25(OH)D ≤ 12 ng/mL (30 nmol/L) [13].

MetS was defined according to the Joint Interim Statement [14] and was considered to be present if at least three (any) of the following characteristics were present: fasting glucose ≥100 mg/dL or drug treatment for elevated glucose; systolic BP ≥130 and/or diastolic BP ≥ 85 mmHg or antihypertensive drug treatment in a patient with a history of hypertension; triglycerides ≥150 mg/dL (1.7 mmol/L) or drug treatment for elevated triglycerides; HDL cholesterol <50 mg/dL (1.3 mmol/L) in women and <40 mg/dL (1.0 mmol/L) in men or drug treatment for reduced HDL-cholesterol; WC ≥88 cm in women and ≥102 cm in men (“European” criteria).

Statistical analysis: Quantitative data are described as the median values and corresponding 25th (P25) and 75th (P75) percentiles. Counts and proportions were reported for categorical variables. Proportions were compared using the chi-square test or *Fisher’s* exact test when appropriate. Mann-Whitney U test was used to compare differences between two independent groups. Multiple linear regression models, with the PTH and 25(OH) D levels as dependent variables, were used to calculate the regression coefficients and their respective 95% confidence intervals (95%CI) of several independent variables, including gender, age, level of education, drinking and smoking habits, UV radiation exposure, physical exercise, Ht, weight, BMI, WC, HC, WHR, WHtR, systolic and diastolic BP, glucose, triglycerides, HDL-cholesterol, total cholesterol, insulin, HOMA, hs-CRP, albumin, calcium, phosphorus and creatinine. The final model was adjusted for age and sex. Unconditional logistic regression models with MetS, its individual components or a “blunted PTH response” as dependent variables were computed, and the odds ratios (OR) and their respective 95% CI were estimated after adjustments for confounding variables.

Results with a two-tailed *p* value <0.05 were considered statistically significant. Statistical analysis was performed using SPSS version 22® software.

## Results

A total of 500 participants (286 women and 214 men) with a median age (P25, P75)) of 53 (41, 67) years [52 (40, 66) years in women and 56 (45, 68) years in men] were included in the present analysis.

The sample characteristics of the 500 participants are presented in Table 2. The median 25(OH)D level was 13.8 ng/mL, with a maximum value of 43.5 ng/mL. According to the VitD adequacy categories, deficiency was present in 37.7% of participants, inadequacy was identified in 47.9% of participants and sufficiency was determined in 14.4% of participants. A seasonal variation of serum 25(OH)D was observed, with significantly higher median values in June–November period compared to the December–May period (*p*˂0.001). The median serum PTH level was 38.1 pg/mL, and hypo- and hyperparathyroidism were present in 0.8% and 9.4% of the participants, respectively.

**Table 2:** Sample characteristics of the 500 participants

	Total	Women	Men	*p* value^a^
Age (years) - median (P25, P75)	53 (41, 67)	52 (40, 66)	56 (45, 68)	0.072
25(OH)D (ng/mL) - median (P25, P75)	13.8 (9.7,17.6)	13.8 (9.7, 17.5)	13.7 (9.9, 17.9)	0.681
25(OH)D (ng/mL) / UV exposure - median (P25, P75)				
Low UV	13.4 (9.3, 17.5)	13.4 (9.3, 16.6)	13.2 (9.0, 17.9)	
High UV	14.3 (10.0, 18.3)^b^	14.1 (9.9, 18.6)	14.5 (10.2, 17.6)	0.603
VitD adequacy - n (%)				
Deficiency	181 (37.7)	102 (37.5)	79 (38.0)	
Inadequacy	230 (47.9)	133 (48.9)	97 (46.6)	
Sufficiency	69 (14.4)	37 (13.6)	32 (15.4)	0.821
PTH (pg/mL) - median (P25, P75)	38.1 (29.8, 49.2)	38.6 (29.8, 50.1)	37.8 (30.0, 47.7)	0.375
“Blunted PTH response” – n (%)				
No	20 (10.8)	16 (15.4)	4 (4.9)	
Yes	165 (89.2)	88 (84.6)	77 (95.1)	0.023
PTH status – n (%)				
Hypoparathyroidism	4 (0.8)	0 (0)	4 (1.9)	
Normal status	449 (89.8)	254 (88.8)	195 (91.1)	
Hyperparathyroidism	47 (9.4)	32 (11.2)	15 (7.0)	0.021
BMI classification - n (%)				
Underweight	6 (1.2)	5 (1.7)	1 (0.5)	
Normal range	159 (31.9)	94 (32.9)	65 (30.5)	
Pre-obese	213 (42.7)	113 (39.5)	100 (46.9)	
Obese	121 (24.2)	74 (25.9)	47 (22.1)	0.242
Metabolic syndrome - n (%)				
No	307 (61.6)	169 (59.3)	138 (64.8)	
Yes	191 (38.4)	116 (40.7)	75 (35.2)	0.213

*SD* standard deviation, *25(OH)D* 25-hydroxyvitamin D, *VitD* vitamin D, *UV* ultraviolet, *low UV* period December–May, *high UV* period June–November, *PTH* parathyroid hormone, *BMI* body mass index

^a^Chi-square test/*Fisher*’s exact test or Mann-Whitney U test p value; ^b^ *p* value <0.001 for the comparison of the mean levels of 25(OH)D according to meteorological periods

A “blunted PTH response” was present in 89.2% of the 185 participants, with serum 25(OH)D levels of less than or equal to 12 ng/mL (Table 3). A “blunted PTH response” was more frequent in men (OR: 4.100; 95%CI: 1.289, 13.036), and the frequency decreased with age (OR: 0.960; 95%CI: 0.930, 0.992). Furthermore, participants with a “blunted PTH response” had significantly higher serum phosphorus levels (OR: 4.590; 95%CI: 1.425, 14.781) and lower hs-CRP levels (OR: 0.493; 95%CI: 0.258, 0.943).

**Table 3:** “Blunted PTH response” characteristics

	Crude OR (95% CI)	OR (95% CI)^a^
Gender		
Women		
Men	3.500 (1.122,10.917)	4.100 (1.289,13.036)
Age	0.964 (0.933,0.996)	0.960 (0.930,0.992)
Height	1.064 (1.006,11.125)	1.002 (0.925,1.085)
BMI	0.936 (0.868,1.010)	0.960 (0.889,1.037)
Systolic BP	0.981 (0.962,1.000)	0.982 (0.958,1.006)
Calcium	1.138 (0.223,5.825)	1.077 (0.211,5.486)
Phosphorus	3.179 (1.173,8.615)	4.590 (1.425,14.781)
Albumin	1.186 (1.050,1.341)	1.138 (0.998,1.299)
Insulin	0.970 (0.909,1.036)	0.969 (0.906,1.036)
HOMA	1.003 (0.979,1027)	1.003 (0.964,1.044)
hs-CRP	0.472 (0.250, 0.892)	0.493 (0.258,0.943)

Some of the variables without significant associations were excluded from the table: education level, drinking and smoking habits, physical exercise, UV exposure, weight, hip and waist circumferences, WHR, WHtR, diastolic BP, glucose, triglycerides, HDL-cholesterol, total cholesterol, creatinine, and 25(OH)D

*OR* odds ratio, *CI* confidence interval, *BMI* body mass index, *BP* blood pressure, *HOMA* homeostatic model assessment, *hs-CRP* high sensitivity C-reactive protein

^a^OR adjusted for gender and age

The associations of the PTH and 25(OH)D levels with various socio-demographic, behavioral and clinical characteristics are presented in Table 4.

**Table 4:** Associations of PTH and 25(OH)D with socio-demographic, anthropometric, clinical and analytical characteristics

	PTH		25(OH)D	
	β (95% CI)^b^	*p* value	β (95% CI)^b^	*p* value
Gender				
Women	^a^		^a^	
Men	−3.331 (−6.415, −0.246)	0.034	0.383 (−0.666,1.433)	0.473
Age (years)	0.320 (0.225,0.415)	<0.001	−0.029 (−0.061,0.003)	0.080
UV exposure				
Low UV	^a^		^a^	
High UV	−0.531 (−2.541,3.603)	0.734	0.940 (−0.105,1.985)	0.078
Physical exercise				
No	^a^		^a^	
Yes	−2.346 (−5.799,1.087)	0.180	1.655 (0.484,2.826)	0.006
BMI	0.475 (0.143,0.807)	0.005	−0.150 (−0.262, −0.037)	0.009
WC	0.184 (0.048,0.320)	0.008	−0.039 (−0.085,0.008)	0.101
Glucose	−2.319 (−7.704,3.066)	0.398	−2.051 (−3.903, −0.199)	0.030
Triglycerides	−1.118 (−3.557,1.322)	0.368	−1.322 (−2.139, −0.505)	0.002
Calcium	−2.098 (−7.923,3.727)	0.479	0.298 (−1.690,2.286)	0.768
Phosphorus	−4.689 (−7.567, −1.812)	0.001	0.245 (−0.748,1.238)	0.628
Creatinine	14.553 (5.267,23.838)	0.002	2.367 (−0.789,5.522)	0.141
PTH			−0.004 (−0.034,0.025)	0.770
25(OH)D	−0.040 (−0.311,0.230)	0.770		

Some of the variables without significant associations were excluded from the table: level of education, drinking and smoking habits, height, weight, hip circumference, WHR, WHtR, systolic and diastolic BP, HDL-cholesterol, total cholesterol, albumin, insulin, HOMA and hs-CRP

*CI* confidence interval, *UV* Ultraviolet, *low UV* period December–May, *high UV* period June–November, *IMC* body mass index, *WC* waist circumference, *PTH* parathyroid hormone serum levels, *25(OH)D* 25-hydroxyvitamin D serum levels

^a^Reference class

^b^β coefficients adjusted for gender and age

The serum levels of PTH and 25(OH)D showed no significant association between them (*p* = 0.770).

The serum PTH levels were significantly lower in men (β: -3.331; 95%CI: -6.415, −0.246) and were positively associated with age (β: 0.320; 95%CI: 0.225, 0.415). In addition, positive associations between PTH and BMI (β: 0.475; 95%CI: 0.143, 0.807), WC (β: 0.184; 95%CI: 0.048, 0.320) and creatinine levels (β: 14.553; 95%CI: 5.267, 23.838) were found. Furthermore, a negative association between PTH and serum phosphorus levels (β: -4.689; 95%CI: -7.567, −1.812) was observed.

The serum 25 (OH)D levels were positively associated with participation in physical exercise (β: 1.655; 95%CI: 0.484, 2.826) and were negatively associated with BMI (β: -0.150; 95%CI: -2.262, −0.037) and serum glucose (β: -2.051; 95%CI: -3.903, −0.199) and triglycerides (β: -1.322; 95%CI: -2.139, −0.505) levels.

MetS was present in 191 participants (38.4%), and its prevalence was slightly higher in women than in men, but without statistical significance (40.7% versus 35,2%, *p* = 0.213). The MetS prevalence significantly increased with age (*p* < 0.001) and with higher HOMA scores (*p* < 0.001) as well as higher insulin (*p* < 0.001) and hs-CRP (*p* = 0.027) levels.

Table 5 shows the associations of PTH and 25(OH)D with MetS and its individual components. The PTH serum levels showed a crude positive association with MetS (OR: 1.016; 95%CI: 1.006, 1.027) and its WC (OR: 1.023; 95%CI: 1.012, 1.034) and BP (OR: 1.022; 95%CI: 1.010, 1.034) features. After adjustments for age and sex (model 1), only the association with the WC feature remained significant. This association remained significant even after further adjustment for 25(OH)D levels (OR: 1.013; 95%CI: 1.001, 1.015). After adjustment for age, sex and BMI (model 2), this association lost statistical significance.

**Table 5:** Associations of PTH and 25 (OH) D with MetS and its components

	PTH		25(OH)D	
	Model 1: OR (95% CI)	Model 2: OR (95% CI)	Model 1: OR (95% CI)	Model 2: OR (95% CI)
MetS	1.004 (0.993,1015)	0.995 (0.983,1.008)	0.957 (0.922,0.993)	0.967 (0.930,1.007)
WC	1.014 (1.002,1.026)^a^	1.002 (0.986,1.017)	0.991 (0.958,1.026)	1.024 (0.980,1.069)
BP	1.006 (0.992,1.020)	1.001 (0.988,1.016)	0.949 (0.912,0.987)	0.954 (0.916,0.993)
Trig	0.996 (0.983,1.008)	0.991 (0.978,1.004)	0.930 (0.892,0.969)	0.937 (0.898,0.978)
HDL	0.998 (0.988,1.009)	0.995 (0.985,1.006)	0.990 (0.959,1.023)	0.997 (0.965,1.030)
Glu	0.998 (0.985,1.010)	0.994 (0.981,1.007)	0.975 (0.936,1.016)	0.990 (0.949,1.033)

*OR* odds ratio, *CI* confidence interval, *PTH* parathyroid hormone serum levels, *25(OH)D* 25-hydroxyvitamin D serum levels, *MetS* metabolic syndrome, *WC* waist circumference component, *BP* blood pressure component, *Trig* triglycerides component, *HDL* HDL cholesterol component, *Glu* glucose component

Model 1: OR adjusted for gender and age; Model 2: OR adjusted for gender, age and body mass index

^a^OR (95%CI) after adjustment for gender, age and 25(OH)D serum level: 1.013 (1.001, 1.025)

We also found a crude negative association between the serum 25 (OH) D levels and MetS (OR: 0.953; 95%CI: 0.921, 0.985) and its BP (OR: 0.951; 95%CI: 0.920, 0.983) and triglycerides (OR: 0.930; 95%CI: 0.893, 0.969) components. After adjustments for age and sex (model 1), all the three associations remained significant. However, after further adjustment for BMI (model 2), the association remained statistically significant only for the BP and triglycerides components of the MetS [(OR: 0.954; 95%CI: 0.916, 0.993) and (OR: 0.937; 95%CI: 0.898, 0.978), respectively].

## Discussion

### VitD status

We found a high prevalence of VitD deficiency or inadequacy (85.6%) in this sample of the Portuguese population. The high prevalence is supported by previously published national evidence (VitD deficiency or inadequacy ranging from 60.3% to 92.7%), although specific population groups were recruited in hospital settings [3, 15, 16]. In addition, according to a recent study, conducted in the city of Porto [17], which included 198 healthy participants, VitD deficiency or inadequacy was present in 48%. In the winter period, these values reached 74%.

Compared with other European [2] and worldwide [18] populations, the median 25(OH)D levels observed in this study were relatively low despite the favorable latitude. The high prevalence of pre-obesity and obesity may have contributed to these figures. Furthermore, VitD intake in the Portuguese population is relatively low [2].

Food fortification, VitD supplementation, sunlight exposure and UV protection habits may also be contributing factors to the differences observed across various countries. Several European countries have adopted measures at the national level to implement VitD supplementation and food fortification policies, but they are not harmonized across Europe [2]. In addition, dietary reference values for VitD intake have been a subject of debate in some countries. In Portugal, there is no legislation of food fortification and VitD supplementation (700–800 IU/day) in adults is only recommended for elderly populations (> 65 years) and subjects with osteoporosis, osteopenia or those with a major risk for osteoporosis [19].

### Functional hypoparathyroidism and the association between VitD and PTH

Despite all of the complex interrelationships, no significant association between PTH and 25(OH)D was observed. This lack of association was previously reported [20] and may be partly explained by a “blunted PTH response” to VitD deficiency [13]. In fact, most participants with Vit D deficiency had a “blunted PTH response” (89.2%). This response cannot be explained by the type of definition of hypovitaminosis D used (IOM) because according to its definition only participants with serum levels of 25 (OH) D lower than 12 ng/mL and with normal serum PTH levels were considered. The “blunted PTH response” may correspond to a protective mechanism of bone mass, through the development of a functional hypoparathyroidism [13]. In addition to parathyroid dysfunction, other factors may contribute to the modification of the PTH response to low VitD levels [21–23], such as age, gender, BMI, kidney dysfunction, smoking, and serum calcium levels. Our results did not show a significant contribution of smoking, BMI and serum levels of calcium and creatinine to the “blunted PTH response”, but we found positive associations with male gender and serum phosphorus levels and negative associations with age and serum levels of hs-CRP. A positive association was already described between PTH and hs-CRP serum levels [24].

In addition to the “blunted PTH response”, other factors may have contributed to the lack of association between the PTH and 25(OH)D levels. The method of measurement strongly influences the VitD and PTH levels and may modify the relationship between them. Specifically, the chemiluminescent immunoassay may have underestimated the 25(OH)D measurements [25]. Regarding the third generation PTH assay, freezing may have contributed to reduced PTH levels [26]. However, previous studies on 25(OH)D showed great stability under freezing conditions [27]. Moreover, despite being a useful biomarker of VitD supply to target tissues, 25(OH)D may not be a good functional marker of the biologically active form, 1α,25(OH)_2_D, relative to PTH regulation. Finally, the upper reference value of normal serum PTH levels (65 pg/mL) that is usually used may not be appropriate as a cut-off point for the definition of high PTH levels [13, 21].

### VitD, PTH and MetS associations

To the best of our knowledge, this is the first national study to evaluate 25(OH)D and PTH levels and their associations with MetS.

According to our data, MetS prevalence was high and increased with age, HOMA score, and insulin and hs-CRP levels (data not shown). These results are supported by previous studies [28].

Our study showed a significant positive association between PTH and age even after adjusting for sex. By contrast, no significant association between 25(OH)D and age was observed. According to a systematic review, PTH levels are positively correlated with age [23]. We also found a significant negative association between serum PTH levels and male gender [29].

Although higher PTH levels have been associated with increased risk of CVD [7], insulin resistance, blood pressure and obesity [8, 9] we did not find a significant association between MetS and PTH levels in the adjusted model. Others have found a positive association between PTH and MetS only among older men [8] and in morbidly obese individuals [9].

We found positive associations of PTH serum levels with BMI, WC and the WC component of MetS. As expected, the association with the WC component of MetS was lost after further adjustment for BMI. The link between PTH levels and increased body fat is supported by the evidence of increased body weight in primary hyperparathyroidism [30] and by the positive association with BMI [31]. This association may be even stronger in visceral adipose tissue [32], explaining the stronger correlation found with WC. PTH may increase adipose mass, especially in the visceral compartment, by increasing the influx of calcium into adipocytes.

The 25 (OH) D levels showed a positive association with participation in physical exercise, which persisted after adjusting for sex and age. Regular outdoor physical activity is associated with higher levels of serum VitD [33], which can be partly explained by higher UV radiation exposure. Unfortunately, in this study, no data were available on the specific type of exercise practiced by the participants in our sample (indoor versus outdoor).

MetS was inversely associated with 25(OH)D levels even after adjusting for age and sex; however, the association was lost after further adjustment for BMI. A recent meta-analysis showed that the prevalence of MetS decreased at higher 25(OH)D concentrations [5].

In addition, the prevalence of hypovitaminosis D is generally increased in adults with CVD, namely in coronary heart disease and heart failure [34], and low 25(OH)D levels are associated with an increased risk of ischemic heart disease, myocardial infarction, and premature death [4]. Furthermore, results from a recent meta-analysis indicate a non-linear decrease in overall mortality as the 25(OH)D levels increase [35]. Low levels of vitamin D have been reported to be associated not only with obesity and insulin resistance but also with glucose intolerance, dyslipidemia, increased renin gene transcription, endothelial dysfunction, proliferation of vascular smooth muscle cells, thrombogenecity and inflammation [5]. However, low levels of vitamin D may be simply secondary to inflammation and other phenomena associated with obesity and insulin resistance.

We found a significant positive association of the 25(OH)D levels with BMI, but not with the WC and WC components of MetS. A bi-directional Mendelian randomization analysis [36] suggested a causal role of obesity for the risk of hypovitaminosis D. However, any causal effect of hypovitaminosis D in obesity is likely to be small. The possible mechanisms for the lower 25(OH) D concentrations in obesity [37] include: lower VitD dietary intake, reduced sunbathing habits due to a decreased willingness to expose the body, sedentary lifestyle and mobility limitations, decreased bioavailability due to sequestration in the adipose tissue and a volumetric dilution effect related to greater body weight.

A negative association of 25(OH)D with glycemia was observed, but not with the glycemic component of MetS. Several longitudinal cohort studies and a recent meta-analysis [38] demonstrated an inverse association between the 25(OH)D levels and increased risk of type 2 diabetes. VitD status can interfere with insulin secretion and insulin resistance. VitD stimulation of β-cell VDRs and local activation of the 1-α-hydroxylase enzyme may contribute to the modulation of pancreatic β-cells calcium influx and insulin synthesis. VitD may also increase insulin sensitivity by increasing insulin receptor expression and by stimulating insulin-induced glucose transport.

A negative association was present between the 25(OH)D levels and triglycerides levels [39] and triglycerides component of MetS. Although the mechanisms are not clear, they may involve metabolism of triglycerides through modulation of the intracellular calcium content of adipocytes and hepatocytes. Hypovitaminosis D-related inflammation and insulin resistance may be other contributing factors.

Although no linear association was found between diastolic or systolic BP and the 25(OH)D levels (data not shown), the 25(OH)D levels showed a significant association with the BP component of MetS. According to the literature, VitD is inversely associated with BP [40], and several mechanisms have been proposed to be related to hypovitaminosis D and hypertension [41], including disruption of the negative endocrine regulation of renin gene expression, secondary hyperparathyroidism, and enhanced vascular tone through direct or indirect dysfunction of the endothelial and vascular smooth muscle cells.

Also, the associations observed between Mets and its individual components and PTH and 25 (OH) D serum levels were generally weak. In fact, this was not surprising as the variables considered in those associations were continuous and therefore the calculated ORs represent the odds of an outcome occurring for an increased unit of PTH or 25(OH)D serum levels.

Lastly, one must acknowledge some additional limitations of this study. Firstly, due to its cross-sectional nature, no causal relation can be inferred from the significant associations observed. Also, and finally, one could expect type I error in the evaluation of the crude associations due to multiple comparisons. Nevertheless, the authors do not expect that this could have occurred in the final model defined for this study, as the number of tested variables was in fact small.

## Conclusion

The present study showed a high prevalence of hypovitaminosis D in a sample of the Portuguese population. Compared with other European and worldwide populations, our median level of 25(OH)D (13.8 ng/mL) is relatively low. The prevalence of hypovitaminosis D was higher in participants with higher BMI and sedentary lifestyles.

The PTH levels showed a significant positive association with BMI, WC and the WC component of MetS, suggesting a possible role in the pathophysiology of obesity. The 25(OH)D levels were negatively associated with BMI, glucose and triglycerides levels as well as with MetS and its BP and triglycerides components, indicating that hypovitaminosis D may contribute to the pathophysiology of MetS.

Considering the low levels and inadequate intake of VitD, the frequency of overweight, and potentially insufficient solar exposure in the Portuguese population, it is crucial to develop national policies to increase awareness of the importance of VitD for health and to develop strategies for the identification of vitamin D deficiency, especially in at-risk groups.

## Abbreviations

25(OH)D: 25-hydroxyvitamin D
BMI: Body mass index
CI: Confidence interval
CVD: Cardiovascular disease
HC: Hip circumference
HOMA: Homeostatic model assessment
hs-CRP: High sensitivity C-reactive protein
Ht: Height
MetS: Metabolic syndrome
OR: Odds ratio
PTH: Parathyroid hormone
SD: Standard deviation
UV: Ultraviolet
VitD: Vitamin D
VitD3: Cholecalciferol
WC: Waist circumference
WHR: Waist-to-hip ratio
WHtR: Waist-to-height ratio
